# Relationship of metabolic syndrome defined by IDF or revised NCEP ATP III with glycemic control among Malaysians with Type 2 Diabetes

**DOI:** 10.1186/s13098-020-00575-7

**Published:** 2020-08-05

**Authors:** Riyadh Saif-Ali, Nor Azmi Kamaruddin, Molham AL-Habori, Sami A. Al-Dubai, Wan Zurinah Wan Ngah

**Affiliations:** 1 Biochemistry Department, Faculty of Medicine, Sana’a University, Sana’a, Yemen; 2 Internal Medicine, UKM Medical Centre, University Kebangsaan Malaysia, Kuala Lumpur, Malaysia; 3 Joint Program of Family Medicine, Post Graduate Studies, Al-Madinah, Saudi Arabia; 4 Genetic Unit, UKM Medical Molecular Biology Institute (UMBI), Kuala Lumpur, Malaysia

**Keywords:** Metabolic syndrome, Type 2 Diabetes, Insulin resistance, Glycemic control

## Abstract

**Background:**

The chronic complications of Type 2 Diabetes (T2D) such as macrovascular disease is amplified with the increase in the number of metabolic syndrome (MetS) risk factors. This research aims to study the relationship of MetS, diagnosed by the International Diabetes Federation (IDF) or revised National Cholesterol Education Programs Adult Treatment Panel III (NCEP ATP III) criteria, with glycemic control, fasting blood glucose (FBG), glycated hemoglobin (HbA1c), C-peptide, and insulin resistance in T2D patients.

**Methods:**

The study is a cross-sectional observational study which, involved 485 T2D patients who are receiving treatment at the University Kebangsaan Malaysia Medical Center (UKMMC), Kuala Lumpur, Malaysia. The MetS among the T2D patients was diagnosed based on IDF and revised NCEP ATP III criteria. C-peptide and HbA1c levels were determined by an automated quantitative immunoassay analyzer and high-performance liquid chromatography, respectively. The MetS factors; FBG, triglyceride, and high-density lipoprotein cholesterol were measured by spectrophotometer.

**Results:**

Application of the IDF and revised NCEP ATP III criteria respectively resulted in 73% and 85% of the T2D subjects being diagnosed with MetS. The concordance of these criteria in diagnosing MetS among T2D patients was low (κ = 0.33, *P *< 0.001). Both IDF and revised NCEP ATP III criteria indicated that T2D patients with 5 MetS factors had higher insulin resistance (*P *= 2.1 × 10^−13^; 1.4 × 10^−11^), C-peptide (*P *= 1.21 × 10^−13^; 4.1 × 10^−11^), FBG (*P *= 0.01; 0.021), and HbA1c (*P *= 0.039; 0.018) than those T2D patients without MetS, respectively.

**Conclusion:**

Although there is a low concordance between IDF and revised NCEP ATP III criteria in the diagnosis of MetS among T2D patients, both criteria showed that T2D patients with 5 MetS factors had higher insulin resistance, C-peptide, FBG, and HbA1c.

## Background

The burden of non-communicable diseases in developing countries is increasing and leading to high mortality rates [1]. Nowadays, Type 2 Diabetes (T2D) is pandemic. The International Diabetes Federation (IDF) estimated that more than 415 million people have diabetes, and this number is expected to reach 642 million by 2040 [2]. The metabolic syndrome (MetS) is a complex disorder with a high socioeconomic impact on global health due to its association with increased morbidity and mortality [3]. The MetS has attracted increased attention due to its significant impact on cardiovascular diseases (CVD) and its high prevalence in T2D patients [4–9]. The MetS can be defined as a cluster of cardio-metabolic dysfunctions which is characterized by the increase in fasting blood glucose (FBG), waist circumference (WC), blood pressure (BP), triglycerides (TG), and reduction in high-density lipoprotein cholesterol (HDL-C) [10, 11].

It is estimated that 20–25% of the world’s adult population suffers from MetS. People with MetS have a threefold increase in the risk of coronary heart disease and stroke and a twofold increased risk of mortality from cardio- and cerebrovascular disease compared with people without the MetS [2, 12]. This global increase in MetS is associated with the worldwide epidemic of obesity andT2D. Obesity and physical inactivity are the driving force for MetS, and a person with MetS has a fivefold relative risk to develop T2D [6, 13–15]. Overweight and obesity lead to adverse metabolic effects on BP, HDL-C, TG, and impaired glucose tolerance (IGT) [16].

The National Cholesterol Education Programs Adult Treatment Panel III (NCEP ATP III) proposed a simple set of diagnostic criteria for MetS based on WC, TG, HDL-C, BP, and FBG levels [17]. In 2005, the IDF modified the MetS definition, which stated that WC is necessary for the diagnosis of MetS along with two or more of the other MetS parameters, including the treatment of the above Mets parameters [18]. In the same year, the American Heart Association and the National Heart, Lung, and Blood Institute revised the NCEP ATP III criteria and affirmed its overall utility and validity and proposed that it continued to be used with minor modifications and clarifications [19] (Table 1).

**Table 1:** Diagnostic criteria of metabolic syndrome

Parameters	Revised NCEP ATP III	IDF
Definition	Any three of the following 5 features	Increased waist circumference^a^ Men ≥ 90 cm, Women ≥ 80 cm along with any 2 of following features
Elevated waist circumference	≥ 102 cm in men ≥ 88 cm in women	
Triglyceride	≥ 1.7 mmol/l or TG treatment	
HDL-C	Men < 1.03 mmol/l or women < 1.29 mmol/l or HDL-C treatment	
Blood pressure	Systolic ≥ 130 mmHg or Diastolic ≥ 85 mmHg or hypertension treatment or previously diagnosed hypertension	
Fasting blood glucose	≥ 5.6 mmol/l or treatment for elevated glucose or previously diagnosed Type 2 Diabetes	

*NCEP ATP III* National Cholesterol Education Programs Adult Treatment Panel III*, IDF* International Diabetes, *FBG* fasting blood glucose Federation, *TG* triglycerides, *HDL*-*C*, high-density lipoprotein cholesterol

^a^Sub-Saharan Africans, Eastern Mediterranean, and Middle East (Arab) populations use European and Ethnic South and Central Americans Use South Asia

In 2009, a meeting between several organizations: International Diabetes Federation Task Force on Epidemiology and Prevention; National Heart, Lung, and Blood Institute; American Heart Association; World Heart Federation; International Atherosclerosis Society; and International Association for the Study of Obesity attempted to unify criteria [20]. In this meeting, the IDF criteria were modified, and it was agreed that WC should not be an obligatory component for the diagnosis of MetS, and three abnormal findings out of 5 would qualify a person for the MetS. However, there is no consensus on the definition of MetS worldwide. Studies revealed that the impact of different definitions of MetS on the risk of future CVD and T2D is discrepant [21, 22].

Several studies have assessed the MetS among normal individuals in different populations, while few studies evaluated the MetS among T2D patients. Taking into consideration, T2D patients who had MetS also have cardiovascular risk factors. Therefore, the diagnosis of MetS in those T2D patients is very important for the detection, prevention, and treatment of the underlying risk factors [23, 24]. This research aims to study the relationship of MetS, diagnosed by the IDF or the revised NCEP ATP III criteria, with glycemic control FBG, HbA1c, C-peptide, and insulin resistance) in T2D patients.

## Methods

### Study design and subjects

The current study was a cross-sectional observational study. Four hundred and eighty-five previously diagnosed T2D patients aged between 30 and 70 years attending the University Kebangsaan Malaysia Medical Center, Kuala Lumpur, Malaysia, were randomly recruited into the study after obtaining their informed consent. Ethical approval was obtained from the National University of Malaysia Research and Ethics Committee.

### Sample and data collection

The WC was measured midway between the lower rib margin and the superior iliac spine at the end of gentle expiration in a standing position. The PB were taken from each patient’s right arm in the seated position by using an Omron IntelliSense Automatic BP Monitor after 10 min rest in a quiet room. Two to three successive BP readings were obtained at 5 min intervals and averaged. Fasting blood (5 ml) was collected from each subject and divided into an EDTA tube for HbA1c measurement, and a plain tube for biochemical investigations. The plain tube was centrifuged for 10 min at 3000×*g* within 30 min of blood collection, and the serum from each sample was separated into 2 Eppendorf tubes and immediately kept at − 20 °C until analysis. The treatment for each participant was collected from the patient data record at the University Kebangsaan Malaysia Medical Center.

### Biochemical analyses and Glycated hemoglobin measurements

Kits for the measurement of glucose, TG, and HDL-C (reference number 10260, 10724, and 10018, respectively) were purchased from Human Company (Human GmbH, Wiesbaden, Germany). The Human company elevated control sera (Humatrol P Reference number 13512) was used as quality control for these parameters. C-peptide was measured in an automated quantitative immunoassay analyzer (Immulite, DPC, Los Angeles, USA) using the IMMULITE C-peptide kit (catalogue number LKPE1). HbA1c levels were determined by high-performance liquid chromatography (VARIANT Hemoglobin A1c Reorder Pack, catalogue number 270-0003, Bio-Rad Laboratories, Inc., Richmond, California, USA) with lyphochek diabetes Bi-level controls (Catalogue number 740) as quality control. Insulin resistance was calculated using the Homeostasis Model Assessment (HOMA2) Calculator v2.2 available from Oxford Center for Diabetes, Endocrinology, and Metabolism. This program used fasting C-peptide or insulin and FBG levels to calculate insulin resistance.

### Assessment of metabolic syndrome

The MetS was diagnosed based on the IDF and revised NCEP ATP III. All subjects included in this study were previously diagnosed with T2D, and therefore FBG was excluded from the five MetS criteria. The MetS in T2D patients was diagnosed according to revised NCEP ATP III that included two or more of the following abnormalities:

1. Central obesity: WC ≥ 102 cm for men, or ≥ 88 cm for women
2. Raised BP: systolic blood pressure ≥ 130 mmHg, diastolic blood pressure ≥ 85 mmHg, or treatment of previously diagnosed hypertension
3. Raised TG levels: ≥ 1.7 mmol/l or specific treatment for this lipid abnormality
4. Reduced HDL-C: men < 1.03 mmol/l, women < 1.29 mmol/l, or HDL-C treatment.

While according to the IDF criteria, the MetS was defined by the presence of central obesity (WC in Asian male ≥ 90 cm and female ≥ 80 cm) together with one of the other MetS factors (BP, TG, and HDL-C) with the same cut off point as revised NCEP ATP III.

### Statistical analysis

The analyses were assessed by SPSS version 11.5 software (SPSS, Inc, Chicago, USA). The FBG, HbA1c, C-peptide, and insulin resistance were log-transformed as they were not normally distributed. Therefore these parameters mean and 95% confidence intervals were transformed back and reported. Cohen’s Kappa (κ) test was used to evaluate the concordance between the IDF and revised NCEP ATP III criteria. The general linear model (adjusted for age, sex, race and family history of diabetes, as covariates) was used to study the correlation of MetS with glycemic control parameters (FBG, HbA1c, C-peptide, and insulin resistance) as a set of dependent variables.

## Results

The T2D patients were on insulin and or oral antidiabetic medications (98%), antihyperlipidemic agents (65%), and antihypertensive medications (64.5%). Three hundred fifty-six (73%) and 415 (85%) out of the 485 T2D patients had MetS when defined by the IDF and revised NCEP ATP III criteria, respectively (Table 2). The application of the harmonizing definition of the MetS resulted in more than 97% of T2D patients with MetS (data not shown). Therefore, this definition could not be included in this study.

**Table 2:** Concordance of International Diabetes Federation and reversed National Cholesterol Education Program criteria in the diagnosis of metabolic syndrome among Type 2 Diabetes patients

	No	% prevalence of MetS in T2D		Concordance (κ, *P* value)
		Revised NCEP ATP III	IDF	
Total	485	85	73	0.33, < 0.001
Men	206	84	62	0.31, < 0.001
Women	279	87	82	0.34, < 0.001
Malaysian Malays	253	88	74	0.35, < 0.001
Men	113	88	63	0.35, < 0.001
Women	140	89	81	0.33, < 0.001
Malaysian Chinese	143	81	67	0.32, < 0.001
Men	65	85	57	0.25, 0.01
Women	78	78	76	0.42, < 0.001
Malaysian Indians	89	85	83	0.24, 0.024
Men	28	68	64	0.28, 0.13
Women	61	93	92	0.08, 0.54

*IDF* International Diabetes Federation, *NCEP ATP III* National Cholesterol Education Programs Adult Treatment Panel III, *T2D* Type 2 Diabetes, *MetS* metabolic syndrome

The IDF and revised NCEP ATP III criteria concurred the diagnosis of MetS in 331 (68%) T2D patients, while 25 (5%) were diagnosed as MetS by the IDF but not by revised NCEP ATP III and 84 (17%) by revised NCEP ATP III criteria but not by IDF (κ 0.33, P < 0.001). The revised NCEP ATP III criteria showed that not much difference in the prevalence of MetS between women with T2D (87%) and men with T2D (84%), while the IDF criteria showed that the prevalence of MetS was higher in women with T2D (82%) than in men with T2D (62%). The revised NCEP ATP III criteria showed that the highest prevalence of MetS was found in Malay patients with T2D (88%) followed by Malaysian Indian (85%) and Malaysian Chinese patients with T2D (81%). While the IDF criteria showed that the highest prevalence of MetS was found among Malaysian Indian patients with T2D (83%) followed by Malay (74%) and the lowest was among Malaysian Chinese patients with T2D (67%). Both criteria showed a higher prevalence of MetS among women with T2D than men with T2D within the three races with low concordance, particularly among Malaysian Indian women (κ = 0.08, P = 0.54) Table 2.

Multivariate analysis of covariance in both IDF and revised NCEP ATP III criteria revealed significant relationships of MetS with glycemic control parameters, Λ’ = 0.865; 0.855, P = 4.8 × 10^−10^; 4.7 × 10^−11^ with powers to detect the relationships were at 0.99998 and 0.99997, respectively. The T2D patients with 5 MetS factors defined by the IDF or revised NCEP ATP III criteria had a significantly higher FBG (*P* = 0.01; 0.021) than T2D patients without MetS (Tables 3 and 4). Both criteria did not show statistical differences between T2D patients with 4 or 3 MetS factors and T2D patients without MetS. HbA1c was higher in T2D patients with 5 MetS factors than T2D patients without MetS (*P* = 0.039; 0.018) in both IDF and revised NCEP ATP III criteria. The IDF criteria showed that T2D patients with 5 MetS factors had a significantly higher HbA1c than T2D patients with 4 or 3 MetS factors (*P* = 0.034, 0.005, respectively).

**Table 3:** The relationship of metabolic syndrome diagnosed by International Diabetes Federation criteria with glycemic control among Type 2 Diabetes patients

Parameters	T2D without MetS (n = 129)	T2D with 3 MetS factors (n = 68)	T2D with 4 MetS factors (n = 146)	T2D with 5 MetS factors (n = 141)
Fasting blood glucose (mmol/l)	7.7 (7.24–8.20)	8.0 (7.33–8.71)	7.9 (7.48–8.39)	8.6 (8.15–9.16)
	P-value	0.516	0.526	0.01 (^b^0.042)
Glycated hemoglobin (%)	7.7 (7.40–8.0)	7.6 (7.18–8.02)	7.6 (7.28–7.83)	8.2 (7.85–8.47)
	P-value	0.69	0.507	0.039 (^a^0.034, ^b^0.005)
C-peptide (pmol/l)	489 (441–542)	569 (493–656)	667 (605–735)	809 (735–893)
	P-value	0.096	2.33 × 10^−5^	1.21 × 10^−13^ (^a^7.1 × 10^−5^, ^b^0.006)
Insulin resistance	2.4 (2.23–2.54)	2.6 (2.39–2.85)	2.9 (2.69–3.03)	3.4 (3.16–3.56)
	P-value	0.110	7.65 × 10^−5^	2.1 × 10^−13^ (^a^5.9 × 10^−5^, ^b^0.0002)

The result presented as geometric mean and 95% confidence interval of mean adjusted to age, sex, race, and history of diabetes

*T2D* Type 2 Diabetes, *MetS* metabolic syndrome

^a^T2D patients with 5 MetS factors versus T2D patients with 3 MetS factors

^b^T2D patients with 5 MetS factors versus T2D patients with 4MetS factors

**Table 4:** The relationship of metabolic syndrome diagnosed by reversed National Cholesterol Education Program criteria with glycemic control among Type 2 Diabetes patients

Parameters	T2D without MetS (n = 70)	T2D with 3 etS factors (n = 136)	T2D with 4 MetS factors (n = 196)	T2D with 5 MetS factors (n = 82)
Fasting blood glucose (mmol/l)	7.8 (7.03–8.23)	8.0 (7.54–8.5)	8.0 (7.62–8.42)	8.8 (8.11–9.49)
	P-value	0.395	0.356	0.021
Glycated hemoglobin (%)	7.6 (7.25–8.07)	7.5 (7.24–7.82)	7.7 (7.49–7.98)	8.4 (7.96–8.80)
	P-value	0.624	0.747	0.018 (^a^0.001, ^b^0.009)
C-peptide (pmol/l)	443 (385–509)	569 (515–628)	688 (633–747)	857 (752–975)
	P-value	0.004	1.5 × 10^−7^ (^c^0.004)	4.1 × 10^−11^ (^a^1.4 × 10^−6^, ^b^0.005)
Insulin resistance	2.3 (2.01–2.49)	2.6 (2.46–2.79)	2.9 (2.77–3.07)	3.5 (3.21–3.78)
	P-value	0.01	3.1 × 10^−6^ (^c^0.01)	1.4 × 10^−11^ (^a^7.6 × 10^−8^, ^b^0.0003)

The result presented as geometric mean and 95% confidence interval of mean adjusted to age, sex, race, and history of diabetes

*T2D* Type 2 Diabetes, *MetS* Metabolic syndrome

^a^T2D patients with 5 MetS factors versus T2D patients with 3 MetS factors

^b^T2D patients with 5 MetS factors versus T2D patients with 4 MetS factors

^c^T2D patients with 4 MetS factors versus T2D patients with 3 MetS factors

C-peptide was significantly higher in T2D patients having 5 MetS factors (*P* = 1.21 × 10^−13^; 4.1 × 10^−11^) or 4 MetS factors (*P* = 2.33 × 10^−5^; 1.5 × 10^−7^) than those T2D patients without MetS using both IDF and revised NCEP ATP III criteria, respectively (Tables 3, 4). The revised NCEP ATP III criteria showed that T2D patients with 3 MetS factors had a significantly higher C-peptide than T2D patients without MetS (*P* = 0.004), whereas the IDF criteria showed no difference (*P* = 0.096). Both IDF and revised NCEP ATP III criteria showed a significantly higher C-peptide in T2D patients who had 5 MetS factors than those who had 4 (*P* = 0.006; 0.005) or 3 MetS factors (*P* = 7.1 × 10^−5^; 1.4 × 10^−6^).

The T2D patients with 5 MetS factors had a higher insulin resistance than T2D patients without MetS (*P* = 2.1 × 10^−13^; 1.4 × 10^−11^), and those who had 3 MetS factors (*P *=5.9 × 10^−5^; 7.6 × 10^−8^) or 4 MetS factors (*P* = 0.0002; 0.0003) when IDF or revised NCEP ATP III criteria were applied, respectively. Both IDF and revised NCEP ATP III criteria showed that insulin resistance was significantly higher in T2D patients with 4 MetS factors (*P* = 7.65 × 10^−5^; 3.1 × 10^−6^) than T2D patients without MetS, respectively. The revised NCEP ATP III criteria showed that T2D patients with 3 MetS factors had a higher insulin resistance than T2D patients without MetS (*P* = 0.01), while IDF criteria did not show a significant association (P = 0.110).

## Discussion

In the present study, the prevalence of MetS among T2D patients was higher according to revised NCEP ATP III criteria compared to IDF, and the concordance between these two criteria was low. However, in German patients with T2D, the IDF criteria showed more MetS than revised NCEP ATP III with a higher concordance (0.69) [25]. In the United Kingdom, the modified NCEP ATP III Criteria (BMI 28.8 kg/m^2^ used instead of WC) showed a higher prevalence than IDF with 0.60 concordance between these criteria [26]. A recent study among Ethiopian patients with T2D showed that the prevalence of MetS according to NCEP ATP III criteria was higher (70%) than IDF (60%) with moderate concordance κ = 0.54 [27]. The low agreement between IDF and revised NCEP ATP III criteria, in this study, is essentially explained by the differences in the contribution of WC to the definition of these two criteria. The IDF stated that WC is necessary for the diagnosis of MetS along with two other MetS factors, while revised NCEP ATP III defined MetS as any three MetS factors. The difference in concordance between MetS diagnostic criteria in different populations is probably due to ethnic characteristics, dietary habits, and lifestyle, thus making it difficult to use a single diagnostic criterion for all populations.

The prevalence of MetS in our Malaysian patients with T2D as defined by revised NCEP ATP III criteria was higher than that reported in Ethiopians [28], Nepalese [29], Iranian [30], sub-Saharan Africans [31]; White, Black, and Mexican Americans [32]. On the other hand, a lower prevalence of MetS 45.8 and 28% was reported from India using NCEP ATP III and IDF criteria, respectively [9], and Ghana 43.83% according to NCEP ATP III and 69.14% by IDF criteria [33]. Similarly, a lower prevalence of MetS was reported in recent studies from Ethiopia 53.5% as defined by the IDF and 66.7% using the NCEP ATP III criteria [34] and from Sri Linka 28.9% and 43.8% using NCEP ATP III and IDF criteria, respectively [35]. A previous study reported a higher prevalence of MetS in Malaysian patients with T2D 96.1% and 84.8% according to NCEP ATP III and IDF definitions, respectively [37].

The increased WC was more frequent in women with T2D (89% and 59%) than men with T2D (68% and 23%) when defined by IDF and revised NCEP ATP III, respectively resulting in a higher prevalence of MetS in women with T2D than men with T2D, which is in agreement with previous studies [26, 29, 35–38]. Also, women with T2D are more likely than men with T2D to have hypertension, low levels of HDL-C, and high levels of TG [39]. Higher prevalence of MetS in female patients with T2D may be due to the higher HDL-C cut-off and lower WC cut-off values in females as compared to males. Hence, more female patients with T2D than male patients with T2D can be recognized as having MetS.

In general, the IDF and revised NCEP ATP III criteria are the most applicable criteria for epidemiological studies and clinical diagnosis of MetS. However, the concordance between these two criteria was low for the diagnosis of MetS among Malaysian patients with T2D. The revised NCEP ATP III criteria utilized American data while the IDF criteria based on accumulated international data. Moreover, there is an ethnic difference in WC, which was considered by IDF. Although the IDF and revised NCEP ATP III criteria were in low concordance for the diagnosis of MetS among Malaysian patients with T2D, there was a similar relationship of MetS with glycemic parameters (insulin resistance, C-peptide, FBG, and HbA1c). The T2D patients with MetS have higher central obesity, which is associated with higher insulin resistance [16]. Accumulation of lipids in the liver and skeletal muscle of T2D patients has been shown to aggravate insulin resistance [40]. As a result, the liver increases glucose production, and muscles utilize less glucose resulting in increased blood glucose. Consequently, beta cells compensate for insulin resistance via an increase in insulin production.

## Conclusion

The relationships of MetS, as defined by either IDF or revised NCEP ATP III criteria, with insulin resistance and poor glycemic control were similar with a low concordance between IDF and revised NCEP ATP III criteria in the diagnosis of MetS among T2D patients. Based on the finding of our study as well as many other studies, it is clear that different definitions of MetS give rise to different prevalence. The difference in the definition of MetS between the two criteria requires more consideration as chronic complications of T2D is amplified with MetS.

## Data Availability

The data are available from the corresponding author on a reasonable request.

## Abbreviations

IDF: International Diabetes Federation
NCEP ATP III: The National Cholesterol Education Programs Adult Treatment Panel III
WHO: World Health Organization
T2D: Type 2 Diabetes
MetS: metabolic syndrome
CVD: cardiovascular diseases
WC: waist circumference
BP: blood pressure
HDL-C: high-density lipoprotein cholesterol
TG: triglycerides
